# Childhood Maltreatment Predicts Specific Types of Dysfunctional Attitudes in Participants With and Without Depression

**DOI:** 10.3389/fpsyt.2021.728280

**Published:** 2021-10-22

**Authors:** Raj Jugessur, Yan Zhang, Xuemei Qin, Mi Wang, Xiaowen Lu, Jinrong Sun, Qiangli Dong, Liang Zhang, Jin Liu, Yumeng Ju, Mei Liao, Ping Wan, Hua Guo, Futao Zhao, Bangshan Liu, Lingjiang Li

**Affiliations:** ^1^National Clinical Research Center for Mental Disorders, Department of Psychiatry, The Second Xiangya Hospital of Central South University, Changsha, China; ^2^Mental Health Institute of Central South University, China National Technology Institute on Mental Disorders, Hunan Technology Institute of Psychiatry, Hunan Key Laboratory of Psychiatry and Mental Health, Changsha, China; ^3^Affiliated WuTaiShan Hospital of Medical College of Yangzhou University, Yangzhou Mental Health Centre, Yangzhou, China; ^4^Zhumadian Psychiatric Hospital, Zhumadian, China

**Keywords:** childhood maltreatment, dysfunctional attitudes, MDD, polyvictimization, depression

## Abstract

**Background:** Studies have shown a strong association between childhood maltreatment (CM) and major depressive disorder (MDD). Dysfunctional attitudes (DAs) play a crucial role in the development of MDD. In this study, we aimed to investigate whether (1) DAs are associated with CM, (2) specific CM types predict specific types of DAs, and (3) higher childhood trauma counts (CTCs) predict more DAs.

**Methods:** One hundred seventy-one MDD participants and 156 healthy controls (HCs) were enrolled for the study. CM was assessed retrospectively with the Childhood Trauma Questionnaire. DAs were evaluated using the Chinese version of the Dysfunctional Attitude Scale–Form A (C-DAS-A). A series of analyses, including multiple analyses of covariance and hierarchical regression analyses, were used in this study to examine the hypotheses.

**Results:** The proportion of CM was 60.2% in the MDD group and 44.2% in the HC group. The 2 × 2 analysis of covariance results showed no interaction effect between CM and MDD on C-DAS-A total score. When the factor scores replaced the C-DAS-A total score, a similar trend was observed. Within the MDD group, emotional abuse (EA) predicted two forms of DAs: self-determination type and overall DAs; physical neglect (PN) was predictive of attraction and repulsion-type DAs. Higher childhood trauma counts significantly predicted more types of DAs in the MDD group.

**Conclusion:** DAs are a trait feature of CM. EA and PN predict specific types of DAs in MDD patients. Higher CTCs predict more DAs in MDD patients.

## Introduction

Childhood maltreatment (CM) is deviant behavior toward an underage that causes harm or entails a risk of causing harm in physical, sexual, and emotional aspect. Several CM forms are recognized: emotional abuse (EA), physical abuse (PA), sexual abuse (SA), and neglect [emotional neglect (EN) and physical neglect (PN)] (1). It constitutes a global threat leading to significant health concerns. Worldwide, one of two children is a victim of any form of CM (2). They lead to severe long-term consequences not limited to work and relationship difficulties, disappointing academic performance, and impaired mental health, including major depressive disorder (MDD) (3–5). Individuals who underwent CM exhibit psychological consequences and disruptions in neurobiological mechanisms; the stress system is affected, and there is impeded brain connectivity, primarily in the frontal brain cortex (6, 7).

Depression is one of the leading causes of psychiatric morbidity globally (8); documentation of its association with CM is not scarce in the medical literature (9, 10). Some reported that MDD was twice likely in individuals with CM (11). CM has a varying effect on depression onset (12), course and response to treatment, and other attributes (10, 13). When considering the individual types of CM, EA increases the risk of depression twice as likely as PA (14); others suggested EN significantly predicts depression, whereas EA correlates with depression severity (15). Under Beck's views of depression, a negative self-schema may be acquired during childhood due to scarring life events. Those are not limited to abuse and neglect. The negative self-schemas remain quiescent unless triggered by stressors (16). Those negative self-schemas, commonly referred to as dysfunctional attitudes (DAs), are ubiquitous negative thought processing styles that affect one's belief about oneself, the world, and the future; they are at the core of depressive pathologies. Several studies thoroughly investigated the impact of DAs in depression patients. They constitute a considerable risk and poorer prognosis of depression (10), as well as being a long-term predictor for relapse (17) and decreased effectiveness to antidepressant therapy (18). The relationship between CM and MDD is moderated by DAs (19, 20). However, not all individuals with DAs will develop depressive disorders, leading us to contemplate whether DAs are a trait resulting from CM. Also, as the individual types of CM have varying effects on depression, could it be possible for the specific CM types to forecast global DAs and specific DAs? Nevertheless, this relationship is unexplored. Only a few studies partly address the question. In a study involving a sample of women, the researcher suggests a significant association between EA and DAs (19). Another researcher suggests a significant association between childhood neglect (CN) and DAs (20). The amount of DAs influences the threshold of an adverse event to onset depression; the higher the DAs grade, the lesser the adverse event's threshold (21, 22). We therefore hypothesized that the more the specific CM types present, the more the DAs.

In this study, we hypothesized that (1) DAs are associated with CM, (2) specific types of CM can predict specific types of DAs, and (3) higher childhood trauma counts (CTCs) can predict more DAs.

## Methods

The set of data used for this study derives from a longitudinal project to scrutinize the psychological and biological mechanisms of MDD (hypothalamic–pituitary–adrenal axis function and magnetic resonance imaging study of trauma-related depression, registration no. ChiCTR1800014591).

One hundred seventy-one participants with MDD were enrolled from inpatient and outpatient departments of the Zhumadian Psychiatric Hospital (Henan, China), and 156 participants were recruited from the local area through flyers for a healthy control (HC) group. The enrolment procedure started in January 2013 and ended in December 2018. An eligibility criterion was set for the two groups, and two well-trained psychiatrists supervised the process.

The enrolment criteria set for the MDD group was as follows: (1) age 18–60 years, (2) diagnosed with MDD and medication-free for not <2 weeks, (3) diagnosis of MDD confirmed by two well-trained psychiatrists using the Structured Clinical Interview for *Diagnostic and Statistical Manual of Mental Disorders, Fourth Edition*, (4) 24-item Hamilton Depression Rating Scale score (HAMD_24_) ≥20 (23), and (5) consent form signed by the patient. The exclusion criteria set was as follows: (1) comorbid Axis I or II or a history of bipolar disorder; (2) history of head injury, neurological disorders, or other internal illnesses; (3) history of substance abuse or dependence except for tobacco dependence; and (4) having suicidal tendencies or ideation. As for the HC group, the inclusion criteria set was as follows: (1) age 18–60 years, (2) HAMD_24_ score <8, and (3) signed consent form by the participant. The HC group's exclusion criteria were as follows: (1) history of any psychiatric disorders; (2) history of substance abuse or dependence except for tobacco dependence; and (3) history of head injury, neurological disorders, or other internal illnesses.

### Measures

#### Depression

A 24-item HAMD_24_ was used to assess depression. It is a commonly used clinician-rated questionnaire (23). The scale was translated by the Shanghai Mental Health Center, and it summed up to have a good reliability and validity in the Chinese community. The scale consists of 24 items: 12 items were rated 0–4, nine items were rated 0–2, and three items were rated 0–3. Hence, the total score ranges from 0 to 75. A cutoff score of at least 20 signifies moderate depression (24).

#### Anxiety

A 14-item Hamilton Anxiety Rating Scale (HAMA_14_) was used to assess anxiety among the participants. It is a clinician-rated questionnaire consisting of 14 items. Each item is rated on a range of 0 (absent) to 4 (severe). The total score ranges from 0 to 56 (25). The Chinese version of the HAMA_14_ summed up to have a good reliability and validity in the Chinese community (26).

#### Dysfunctional Attitudes

The Dysfunctional Attitude Scale was used to assess cognitive vulnerabilities. The Chinese version of the Dysfunctional Attitude Scale–Form A (C-DAS-A) was used for the study. It is a self-reporting scale designed to evaluate DA's rectitude (27). This scale has good reliability and validity in Chinese MDD samples (28, 29). The Chinese version of the scale includes 40 items and encompasses eight subscales. The total score ranges from 40 to 280, with higher the total score, the more DAs. The eight subscales are vulnerability, attraction and repulsion, perfectionism, compulsion, seeking applause, dependence, self-determination attitude, and cognition philosophy (28). More details about the C-DAS-A questionnaire and the nature of the several factors involved can be found in our other articles (30, 31).

#### Childhood Maltreatment

CM was assessed using the Childhood Trauma Questionnaire (CTQ). It is a retrospective assessment tool consisting of five factors for maltreatment, and it is evaluated through 28 items in the questionnaire. It accounts for maltreatment before the age of 16 years, and it is summed up to have good reliability and validity in the Chinese community. The five factors for CM assessed are EA, PA, SA, EN, and PN. Participants were identified as positive for CM if any one of these factors exceeded their cutoff score as mentioned: EA > 12, PA > 9, SA > 7, EN > 14, and PN > 9 (32–34). The CTC was defined as the sum of the total CTQ factors exceeding their respective cutoff scores. Its minimal score is therefore 0, whereas the maximal score is 5.

### Data Analytic Plan

SPSS version 25.0 was used for the analytic procedure and a *p* = 0.05 (two-tailed) for statistical significance. χ^2^ tests and independent *t*-tests were used to check for group differences for categorical variables and continuous variables, respectively, in the MDD and HC groups. To test our first hypothesis, that is, DAs are associated with CM; we used a 2 × 2 analysis of covariance (ANCOVA) of the diagnosis and CM on C-DAS-A total score with age, sex, and education as covariates; *post-hoc* analyses followed it. The same procedure was repeated with the eight C-DAS-A subscale scores as the dependent variable.

For our second hypothesis, a hierarchical regression analysis was used to estimate the different CM types' influence magnitude on C-DAS-A total score first. Then, the eight various C-DAS-A subscale scores replaced the C-DAS-A total score. The procedure was run in the two groups, MDD and HC groups, independently. Afterward, we assessed whether higher CTCs lead to more DA, that is, our third hypothesis, by running a hierarchical regression analysis of CTC on C-DAS-A total score followed by its substitution with the eight different DAS factor scores. The process was run separately in the MDD and HC groups.

## Results

### Demographic/Clinical Information/Prevalence of CM, CM Types, and CTC

Three hundred twenty-seven participants fulfilled the eligibility criteria, including 171 MDD and 156 HC participants. The mean age of the MDD group (35.06 years) was higher than the HC group (34.62 years). The average years of schooling in the MDD group (10.23 years) was lower than in the HC group (11.12 years). The male proportion was also lower in the MDD group (male 43.9%) than that in the HC group (male 45.5%).

Within the MDD group, the mean age at onset of depression was 31.74 years. The average number of episodes of depression was 2.03. There was no statistical significance in age and gender between MDD and HC groups (*p* > 0.05). Both HC/CM^+^ and HC/CM^−^ had more years of education compared to MDD/CM^+^ and MDD/CM^−^. The MDD/CM^+^ group had higher mean scores in HAMD_24_, HAMA_14_, C-DAS-A total, and CTQ total than the MDD/CM^−^ group, and they were all significant (*p* < 0.001). Clinical and demographic characteristics are shown in Table 1.

**Table 1:** Demographics and clinical information of major depressive disorder (MDD) and healthy control (HC) groups.

**Item**	**MDD**		***p*_**1**_**	**HC**		***p*_**2**_**	***P*_**3**_**
	**CM^**+**^ (*n* = 103) Mean ± SD**	**CM^**−**^ (*n* = 68) Mean ± SD**		**CM^**+**^ (*n* = 69) Mean ± SD**	**CM^**−**^ (*n* = 87) Mean ± SD**		
Age (years)	34.61 ± 9.42	35.74 ± 10.08	0.459	36.09 ± 9.30	33.45 ± 8.96	0.074	0.672
Gender (male/female)	41/62	34/34	—	28/41	43/44	—	0.387
Education (years)	9.55 ± 3.29	11.26 ± 3.52	**0.001**	10.26 ± 3.37	11.80 ± 3.68	**0.008**	**0.024**
HAMD_24_	32.01 ± 7.67	31.00 ± 6.80	0.379	1.79 ± 1.89	1.06 ± 1.61	**0.011**	**0.000**
HAMA_14_	18.54 ± 6.32	17.96 ± 6.17	0.551	1.55 ± 2.14	1.01 ± 1.73	0.084	**0.000**
C-DAS-A total	159.11 ± 25.50	149.37 ± 29.13	**0.022**	133.13 ± 22.83	117.40 ± 22.72	**0.000**	**0.000**
CTQ	48.12 ± 9.20	32.16 ± 4.46	**0.000**	44.94 ± 8.81	30.64 ± 4.10	**0.000**	**0.000**
Episodes	1.98 ± 1.18	2.10 ± 1.53	0.556	—	—	—	—
Onset age (years)	31.35 ± 10.09	32.36 ± 10.30	0.537	—	—	—	—
Current history	5.20 ± 11.00	3.50 ± 3.16	0.217	—	—	—	—
Total history	41.14 ± 50.01	42.32 ± 53.13	0.883	—	—	—	—

*Data are presented as mean ± SD. Bold values indicate statistical significance. MDD, major depressive disorder; HC, healthy control; CM, childhood maltreatment; BMI, body mass index; HAMD_24_, 24-item Hamilton Rating Scale for Depression; HAMA_14_, 14-item Hamilton Anxiety Rating Scale; p_1_, statistical significance of MDD group; p_2_, statistical significance for HC group; p_3_, statistical significance of MDD and HC groups*.

The prevalence of CM types and CTC is shown in Table 2. The prevalence of CM in our sample was 52.5%, whereas 60.2% of the MDD group reported CM. PN (43.1%) had the highest prevalence in the sample, in the MDD (49.7%) and the HC (35.9%) groups. SA (8.8%) was the least prevalent form of CM among the MDD group, whereas EA (3.2%) was the least common in the HC group.

**Table 2:** Prevalence of childhood maltreatment and factors in the sample.

		**MDD**		**HC**		**MDD+HC**		**χ^**2**^**	***p***
		***n***	**%**	**n**	**%**	**n**	**%**		
Childhood maltreatment		103	60.2	69	44.2	172	52.6	8.380	**0.004**
Emotional abuse		16	9.4	5	3.2	21	6.4	5.137	**0.023**
Physical abuse		16	9.4	8	5.1	24	7.3	2.145	0.143
Sexual abuse		15	8.8	10	6.4	25	7.6	0.664	0.442
Emotional neglect		55	32.2	30	19.2	85	26.0	7.093	**0.008**
Physical neglect		85	49.7	56	35.9	141	43.1	6.344	**0.012**
CTC	0	68	39.8	86	55.1	154	47.1	15.596	**0.012**
	1	41	24.0	40	25.6	81	24.8		
	2	46	26.9	22	14.1	66	20.8		
	3	10	5.8	5	3.2	15	4.6		
	4	6	3.5	2	1.3	8	2.4		
	5	0	0.0	1	0.6	1	0.3		

*Bold values indicate statistical significance. MDD, major depressive disorder; HC, healthy control; CTC, Childhood trauma count; p, statistical significance of χ^2^ test*.

Most participants (52.9%) reported at least one type of CM from the sample, whereas 28.1% reported at least two types of CM, 7.3% reported at least three types of CM, 2.7% reported at least four types of CM, and 0.3% reported all types of CM.

A higher proportion of participants in the MDD group reported having experienced maltreatment in the past. Similarly, the proportion for the subtypes of CM was higher in the MDD group than that in the HC group. As for CTC, the HC group's proportion was higher than the MDD group for scores 0, 1, and 5, whereas the reverse was observed with CTC scores 2, 3, and 4.

### Effect of Diagnosis and CM on C-DAS-A Total and Subscale Scores

Table 3 shows the results of a 2 × 2 ANCOVA (factor 1: diagnosis and factor 2: CM) on C-DAS-A total and subscale scores with age, gender, and education as covariates. No significant two-way interaction effect of CM and diagnosis was found for C-DAS-A total score while controlling for covariates (*F* = 1.20, *p* = 0.275, partial η^2^ = 0.004). Therefore, an analysis of the main effects and the Bonferroni *post-hoc* test were performed for CM and diagnosis (35). The main effect of CM showed a statistically significant difference in unweighted adjusted marginal mean (36, 37) C-DAS-A total score for those who had CM (145.57) vs. those without CM (134.03) was 11.542 [95% confidence interval (CI), 5.83–17.25; *p* < 0.001]. As for the main effect of diagnosis, it showed a statistically significant difference in unweighted adjusted mean C-DAS-A total score for those of the MDD group (154.10) vs. those of the HC group (125.50). The difference was 28.60 (95% CI, 23.04–34.16; *p* < 0.001).

**Table 3:** Analysis of covariance (ANCOVA) of C-DAS-A total and subscale scores with age, gender, and education controlled.

**C-DAS-A**	**MDD**		**HC**		***F*_**1**_**	***p*_**1**_**	**Main effects of diagnosis**		**Main effects of CM**		**Interaction effects (CM and diagnosis)**	
	**CM^**+**^ (*n* = 103) Mean ± SD**	**CM^**−**^ (*n* = 68) Mean ± SD**	**CM^**+**^ (*n* = 69) Mean ± SD**	**CM^**−**^ (*n* = 87) Mean ± SD**			***F*_**2**_**	***p*_**2**_**	***F*_**3**_**	***p*_**3**_**	***F*_**4**_**	***p*_**4**_**
Total score	159.11 ± 25.5	149.37 ± 29.13	133.13 ± 22.83	117.4 ± 22.72	24.71	** <0.001**	102.44	** <0.001**	15.83	** <0.001**	1.20	0.275
Vulnerability	18.59 ± 4.67	17.19 ± 4.34	15.64 ± 4.81	13.92 ± 3.81	9.83	** <0.001**	38.97	** <0.001**	8.54	**0.004**	0.10	0.754
Attraction and repulsion	19.18 ± 5.25	16.90 ± 6.05	14.65 ± 5.10	11.30 ± 4.24	20.51	** <0.001**	74.73	** <0.001**	20.89	** <0.001**	0.52	0.471
Perfectionism	18.91 ± 5.73	18.38 ± 5.50	16.10 ± 4.59	13.83 ± 4.70	8.92	** <0.001**	38.92	** <0.001**	4.80	**0.029**	2.51	0.114
Compulsion	19.26 ± 4.75	18.44 ± 4.21	16.29 ± 4.12	16.10 ± 3.59	9.71	** <0.001**	29.17	** <0.001**	0.05	0.828	0.68	0.410
Seeking applause	20.44 ± 5.44	18.06 ± 4.95	17.30 ± 4.51	14.48 ± 4.55	12.57	** <0.001**	36.03	** <0.001**	18.01	** <0.001**	0.41	0.522
Dependence	19.68 ± 4.65	19.34 ± 4.80	16.52 ± 3.96	14.13 ± 4.48	14.95	** <0.001**	66.28	** <0.001**	5.22	**0.023**	4.55	**0.034**
Self-determination attitude	22.37 ± 5.88	21.74 ± 5.21	18.81 ± 5.03	17.08 ± 4.60	10.76	** <0.001**	52.94	** <0.001**	5.67	**0.018**	0.53	0.467
Cognition philosophy	20.67 ± 5.32	19.32 ± 5.21	17.81 ± 4.06	16.56 ± 5.15	7.94	** <0.001**	22.73	** <0.001**	2.29	0.131	0.34	0.853

*Data are presented as mean ± SD. Bold values indicate statistical significance. HC, healthy control; CM, childhood maltreatment; C-DAS-A, Chinese version of Dysfunctional Attitude Scale–Form A; F_1_, F test value for corrected model; p_1_, statistical significance of corrected model; F_2_, F test value for main effects of MDD; p_2_, statistical significance for main effects of MDD; F_3_, F test value for main effect of CM; p_3_, statistical significance of main effect of CM; F_4_, F test value for interaction effect between CM and MDD; p_4_, statistical significance of interaction effect between CM and MDD*.

There was no statistically significant two-way interaction of CM and diagnosis while controlling for covariates, on C-DAS-A subscale scores, except for C-DAS-A dependence. These statistically significant interactions were interpreted through analysis of main effects and Bonferroni *post-hoc* analyses of CM and diagnosis. The main effect of CM had statistically significant adjusted marginal means in the following C-DAS-A subscales: vulnerability (1.497, *p* = 0.009), attraction and repulsion (2.717, *p* <0.001), perfectionism (1.321, *p* = 0.029), seeking applause (2.408, *p* < 0.001), and self-determination (1.440, *p* = 0.018). No statistically significant adjusted marginal mean scores were observed for the main effect of CM in C-DAS-A compulsion and cognition philosophy subscales (*p* > 0.131). As for the main effects of diagnosis group, there was a statistically significant adjusted marginal means in all of the eight subscale scores of C-DAS-A (*p* < 0.001).

As for C-DAS-A dependence, a statistically significant two-way interaction of CM and diagnosis was present while controlling for covariates (*F* = 4.55, *p* = 0.034, partial η^2^ = 0.014). Therefore, an analysis of simple main effects for CM and diagnosis was performed using a Bonferroni adjustment and being accepted at the *p* < 0.025 level for both CM and diagnosis (37–40). The effects of diagnosis for CM^+^ (*F* = 18.635, *p* < 0.001, partial η^2^ = 0.135) and CM^−^ (*F* = 50.832, *p* < 0.001, partial η^2^ = 0.055) were both statistically significant. The effect of CM for the MDD group (*F* = 0.021, *p* = 0.886, partial η^2^ = 0.000) was not statistically significant, unlike that for the HC group (*F* = 9.588, *p* = 0.002, partial η^2^ = 0.029).

### Hierarchical Regression Analysis of CM Types on C-DAS-A Total and Subscale Scores

A hierarchical regression analysis was run at three levels to determine if CM types improved the prediction of C-DAS-A total and subscale scores in the MDD and HC groups. At level 1: age, gender, and education; level 2: HAMA_14_ and HAMD_24_; and level 3: EA, PA, SA, EN, and PN were included for the hierarchical regression analysis in the HC group, whereas in the MDD group, two supplementary items were added to level 2: duration of current episode and episode counts. As six participants had missing records of the HAMA_14_ data, they were removed from this investigation leading to a new sample size of 168 for the MDD group and 153 for the HC group.

The hierarchical regression analysis of CM types on C-DAS-A total and subscale scores within the MDD group is shown in Table 4. Within the MDD group, the CM types' addition to the model led to a statistically significant Δ*R*^2^ of 7.9% (*p* = 0.015) with an EA standard coefficient of 0.249 in C-DAS-A total score. There was a statistically significant Δ*R*^2^ of 8.2% (*p* = 0.015) in the C-DAS-A attraction and repulsion score and a statistically significant PN standard coefficient (0.276). In comparison, in the C-DAS-A self-determination, the EA (0.262) was statistically significant, with a Δ*R*^2^ of 6.9% (*p* = 0.027).

**Table 4:** Hierarchical regression analysis of childhood maltreatment types on C-DAS-A total and subscale scores in the MDD group (*n* = 168).

**C-DAS-A**	**Δ*R*^**2**^**	***p* for Δ*R*^**2**^**	**Standard coefficient**				
			**Emotional abuse**	**Physical abuse**	**Sexual abuse**	**Emotional neglect**	**Physical neglect**
Total score	0.079	**0.015**	**0.249**	−0.107	−0.038	0.044	0.119
Vulnerability	0.054	0.101	**0.230**	−0.094	−0.006	0.048	0.059
Attraction and repulsion	0.082	**0.015**	0.113	−0.075	−0.009	−0.015	**0.276**
Perfectionism	0.019	0.685	0.118	−0.054	−0.027	0.075	0.000
Compulsion	0.032	0.334	0.131	−0.005	−0.004	−0.048	0.124
Seeking applause	0.049	0.126	0.165	−0.003	−0.003	0.114	0.009
Dependence	0.031	0.400	0.180	−0.114	0.034	−0.121	0.088
Self-determination attitude	0.069	**0.027**	**0.262**	−0.155	−0.023	0.001	0.118
Cognition philosophy	0.052	0.115	0.134	−0.064	−0.125	0.162	−0.050

*Bold values indicate statistical significance. The ΔR^2^ indicates the changes R^2^ of the model from level 2 to level 3. The three hierarchies of the regression model were as follows: level 1: age, sex, education; level 2: HAMA_14_, HAMD_24_, duration of current episodes, episode counts; level 3: childhood maltreatment types. C-DAS-A, Chinese version of Dysfunctional Attitude Scale–Form A*.

Table 5 shows the hierarchical regression analysis of CM types on C-DAS-A total and subscale scores within the HC group. Only PN (0.216) was statistically significant, with a change in *R*^2^ of 7.7% (*p* = 0.033) observed in C-DAS-A seeking applause.

**Table 5:** Hierarchical regression analysis of childhood maltreatment types on C-DAS-A total and subscale scores in the HC group; (*n* = 153).

**C-DAS-A**	**Δ*R*^**2**^**	***p* for Δ*R*^**2**^**	**Standard coefficient**				
			**Emotional abuse**	**Physical abuse**	**Sexual abuse**	**Emotional neglect**	**Physical neglect**
Total score	0.088	**0.007**	0.048	−0.100	0.044	0.117	0.135
Vulnerability	0.061	0.090	0.151	−0.063	0.087	0.141	−0.032
Attraction and repulsion	0.090	**0.011**	0.107	−0.010	−0.044	0.220	0.089
Perfectionism	0.060	0.089	0.127	−0.105	0.077	0.136	0.037
Compulsion	0.016	0.749	−0.007	−0.130	0.031	0.021	0.067
Seeking applause	0.077	**0.033**	0.139	−0.085	0.057	−0.026	**0.216**
Dependence	0.054	0.098	0.143	−0.009	0.031	−0.113	0.198
Self-determination attitude	0.039	0.269	−0.115	−0.055	0.128	0.052	0.168
Cognition philosophy	0.051	0.123	0.222	−0.078	0.130	0.156	−0.430

*Bold values indicate statistical significance. The ΔR^2^ indicates the changes R^2^ of the model from level 2 to level 3. The three hierarchies of the regression model were as follows: level 1: age, sex, education; level 2: HAMA_14_, HAMD_24_; level 3: childhood maltreatment types. C-DAS-A, Chinese version of Dysfunctional Attitude Scale–Form A; HC, healthy control*.

### Hierarchical Regression Analysis of CTC on C-DAS-A Total and Subscale Scores

A hierarchical regression analysis was run to find CTC's predictability on C-DAS-A total and subscale scores in both the MDD (*n* = 168) and the HC (*n* = 153) groups. At level 1: age, gender, and education; level 2: HAMA_14_ and HAMD_24_; and level 3: CTC were included for the hierarchical regression analysis in the HC group, whereas in the MDD group, two supplementary items were added to level 2: duration of current episode and episode counts. The results are shown in Table 6. C-DAS-A total score had a significant predicted Δ*R*^2^ of 3.8% (*p* = 0.010, β = 0.213) in the MDD group. Other C-DAS-A subscales that showed a significant Δ *R*^2^ were as follows: vulnerability (Δ*R*^2^ = 2.5%, *p* = 0.042, β = 0.171), attraction and repulsion (Δ*R*^2^ = 5.4%, *p* = 0.002, β = 0.253), and seeking applause (Δ*R*^2^ = 3.4%, *p* = 0.014, β = 0.202). In the HC group, the C-DAS-A attraction and repulsion score (Δ*R*^2^ = 2.7%, *p* = 0.036, β = 0.167) led to a statistically significant rise with the addition of CTC to the investigation.

**Table 6:** Hierarchical regression analysis of childhood trauma count on C-DAS-A total and subscale scores in the MDD (*n* = 168) and the HC (*n* = 153) groups.

**C-DAS-A**	**MDD**			**HC group**		
	**(*****n*** **=** **168)**			**(*****n*** **=** **153)**		
	**Δ*R*^**2**^**	***p* for Δ*R*^**2**^**	**Standard coefficient for CTC**	**Δ*R*^**2**^**	***p* for Δ*R*^**2**^**	**Standard coefficient for CTC**
Total score	0.038	**0.010**	**0.213**	0.011	0.156	0.108
Vulnerability	0.025	**0.042**	**0.171**	0.002	0.555	0.048
Attraction and repulsion	0.054	**0.002**	**0.253**	0.027	**0.036**	**0.167**
Perfectionism	0.010	0.206	0.107	0.002	0.629	0.039
Compulsion	0.012	0.138	0.121	0.000	0.829	−0.017
Seeking applause	0.034	**0.014**	**0.202**	0.014	0.137	0.121
Dependence	0.001	0.681	0.035	0.007	0.280	0.084
Self-determination attitude	0.019	0.064	0.150	0.008	0.255	0.090
Cognition philosophy	0.005	0.363	0.076	0.000	0.874	0.012

*Bold values indicate statistical significance. The three hierarchies of the regression model in the MDD group were as follows: level 1: age, sex, education; level 2: HAMA_14_, HAMD_24_, duration of current episode, episode counts; level 3: childhood maltreatment types. The three hierarchies of the regression model in the HC group were as follows: level 1: age, sex, education; level 2: HAMA_14_, HAMD_24_; level 3: childhood trauma counts. The ΔR^2^ indicates the changes R^2^ of the model from level 2 to level 3. C-DAS-A, Chinese version of Dysfunctional Attitude Scale–Form A; HC, healthy control; CTC, childhood trauma count*.

## Discussion

Up to our knowledge, this study is among the few to investigate DAs as trait features of CM. The reported prevalence rate of CM among the depressed participants and HCs was 60.2% and 44.2%, respectively. Our reported prevalence rate was much higher than a meta-analysis conducted in 2017. However, the meta-analysis reported a comparatively lower prevalence rate of 45.6% among depressed participants (15). This discrepancy could be because our study was restricted to one region, and we had a smaller sample size. The disparity suggests that CM could be more frequent in some regions. The reported prevalence of specific CM types was comparatively lower, except for PN, within the depressed participants (EA: 9.4% vs. 36.7%; PA: 9.4% vs. 27.6%; SA: 8.8% vs. 25.3%; EN: 32.3% vs. 43.2%; PN: 49.7% vs. 36.2%). Given our study's regional concept, China's rapid economic development meant parents have less time to interact with their children physically. As stated by the social development theorist Vygotsky, children do not develop in isolation; the lack of social interaction suffered by the children neglected by their caregivers constitutes a social impediment for their cognitive development.

Beck's cognitive theory of depression proposed that a negative self-schema is present before the onset of depression. Those cognitive distortions are results of adverse childhood experiences. They remain dormant until triggered by stressors (16, 41). By demonstrating cognitive differences between individuals who underwent CM and the depressed participants, this study provides essential support to Beck's cognitive theory of depression. We showed that CM predicts DAs in both participants with and without depression. Thereby, we understand that CM predicts some amount of DAs, which remain latent. We shared a similar tenet with a study about mood induction. They showed that DAs remain latent unless activated (42). We also shared similar results with a survey of 155 participants; they found a significant association between DAs and CM (43). However, only healthy participants with a comparatively lower mean age were involved in that study.

DAs are molded through adverse experiences starting from childhood. CM is among the risk factors for cognitive vulnerabilities (44). Maltreated children make inferences in trying to understand maltreatment events. With the repetition of those events, the children can develop DAs by negative cognitive structuring and faulty information processing. Ultimately, depression is the result when those are triggered (21, 44, 45). Studies have found that EA and EN had a strong association with DAs (46, 47). They are also predictive of future depressive episodes (48–50), mediated by DAs (43). Our study is on similar lines. We found that individuals with EA were likely to develop more DAs of self-determination attitude type and overall DAs among the depressed participants. It is possible that those two types of DAs might influence the pathway from EA to depression. Individuals with DAs of self-determination attitude type are those with the thought of casting one's values in comparison with others (e.g., “If I do not do as well as other people, it means I am an inferior human being”) (28). A group of researchers shared similar findings; they discussed the relationship between EA and depression mediated by DAs (19).

Failure to cater to a child's basic needs by caregivers, either deliberately or unknowingly, defines CN. The child is deprived of basic needs, safety, supervision, medical care, physical requirements, and emotional support (1). CN includes PN and EN. It is the most prominent form of CM worldwide, and its high prevalence can be seen in our study. Approximately one in six children will experience CN (51). Studies have shown that CN impedes the development of the corpus callosum areas (52), and those alterations correlate with depression (53–55). An interesting result from our research indicated that PN is bound to more DAs: attraction and repulsion-type DA in the depressed and seeking applause-type DA in the non-depressed. However, a study of 155 healthy participants with a mean age of 18.8 years found that physical maltreatment was not related to DAs. In their research, physical maltreatment englobed PA and PN. Their selection of only healthy participants makes it difficult for us to weigh up our depression group results. Nevertheless, we could compare the findings of our control group. The reason behind this discrepancy could be the merging of PA and PN into one category (43), as PA was reported not to be associated with DAs (19). CN was found to be predictive of DAs among depressed participants in a study (20), partly supporting our findings for PN.

Exposure to one form of trauma in childhood potentially elevates the risk of experiencing several forms of trauma over time. Polyvictimization is the term used to describe individuals who experienced potentially traumatic events such as the known components of CM, bullying, and witnessing adverse events such as parent substance abuse, domestic violence, and others (56, 57). It is a robust predictor of short- and long-term mental health problems not limited to depression (14, 58). Like a few, we also used the CTQ to assess an aspect of polyvictimization. They found a “dose-dependent” relationship between collective CM types and the odds of being diagnosed with depression (59, 60). Our study added a new scope in polyvictimization; we further assessed which type of DA is more likely in depressed and non-depressed individuals. With increased CTCs, DAs of vulnerability type, attraction and repulsion type, and seeking applause type and overall DAs were predicted in depressed patients. In those without depression, increased CTCs predicted DAs of attraction and repulsion type. Our results were in line with the titration model of the cognitive vulnerability. It states that a lesser threshold of adverse events is required when more negative cognitive styles are present to onset depression (21, 22).

## Limitations

A couple of limitations concerning this study should be noted. First, the nature of our research is a hurdle to make reverse causality inferences. We could not show the direct causality of DAs associated with CM and account for time exposed to CM. Second, the retrospective assessment of CM using the CTQ is subject to recall biases. Also, some forms of CM such as SA might be underreported in fear of shame and social detriment. Third, polyvictimization is best assessed using the Juvenile Victimization Questionnaire (JVC) (57). As most of the JVC components overlapped with the CTQ, we adopted the latter for our study's purposes. Two researchers endorsed the same method (59, 60). They assessed polyvictimization by grading the severity of the individual CTQ factors. We used a dichotomous format for each factor of the CTQ; either presence or absence could be the outcome, ignoring the severity of the CTQ factors.

## Conclusions

Our group of researchers brought up the novel idea to examine the type of DAs predicted by CM types, and we are the only to explore the types of DA predicted by higher CTCs. In summary, our study provided new insights into the clinical field. Specific types of DAs might influence the relationship between MDD and CM. Furthermore, we also concluded that the higher the CTCs, the more DA types in participants with and without depression. Screening and prevention of CM by related authorities, caregivers, medical professionals, and parents are imperative to break the chain. EA or PN typically deserves better attention; they may be potential markers to screen for depression. Research has shown that psychotherapy alone or in combination with antidepressants is best suited in depressed patients who underwent CM (61). Cognitive–behavioral therapy (CBT), personalized trauma–focused CBT, and child–parent psychotherapy are recommended. The forms of DA associated with depression found in our study should to be focused on to address ongoing or future depressive episodes.

## Data Availability Statement

The raw data supporting the conclusions of this article will be made available by the authors, without undue reservation.

## Ethics Statement

The studies involving human participants were reviewed and approved by the Ethics Committee of the Second Xiangya Hospital of Central South University and Ethic Committee of the Zhumadian Psychiatric Hospital. The patients/participants provided their written informed consent to participate in this study.

## Author Contributions

LL, YZ, and BL co-designed the topic. BL, JS, MW, XL, QD, LZ, JL, YJ, PW, HG, FZ, and YZ are responsible for participant recruitment and data collection. RJ and BL co-conducted the statistical analyses. RJ wrote the initial draft of the manuscript. BL contributed important revisions to the manuscript. All authors contributed to the article and approved the submitted version.

## Funding

This study was supported by the National Science and Technologic Program of China (2015BAI13B02), the Defense Innovative Special Region Program (17-163-17-XZ-004-005-01), the National Natural Science Foundation of China (81171286, 91232714, and 81601180). The funding sources had no role in the study design, data collection and analysis, interpretation of the data, preparation and approval of the manuscript, and decision to submit the manuscript for publication.

## Conflict of Interest

The authors declare that the research was conducted in the absence of any commercial or financial relationships that could be construed as a potential conflict of interest.

## Publisher's Note

All claims expressed in this article are solely those of the authors and do not necessarily represent those of their affiliated organizations, or those of the publisher, the editors and the reviewers. Any product that may be evaluated in this article, or claim that may be made by its manufacturer, is not guaranteed or endorsed by the publisher.

## References

[1] World Health Organization. Preventing Child Maltreatment: A Guide to Taking Action and Generating Evidence. Geneva: World Health Organization (2012).

[2] Hillis S Mercy J Amobi A Kress H. Global prevalence of past-year violence against children: a systematic review and minimum estimates. Pediatrics. (2016) 137:e20154079. 10.1542/peds.2015-4079 26810785 PMC6496958

[3] Bradshaw J Hooper C-A. Child maltreatment. In: Bradshaw J. editor. The Well-Being of Children in the UK. Bristol: The Policy Press (2011). p. 191–211.

[4] Chapman DP Whitfield CL Felitti VJ Dube SR Edwards VJ Anda RF. Adverse childhood experiences and the risk of depressive disorders in adulthood. J Affect Disord. (2004) 82:217–25. 10.1016/j.jad.2003.12.013 15488250

[5] Abbasi MA Saeidi M Khademi G Hoseini BL Moghadam ZE. Child maltreatment in the worldwide: a review article. Int J Pediatr. (2015) 3:353–65. 10.22038/ijp.2015.3753

[6] Cicchetti D Toth SL. Child maltreatment. Annu Rev Clin Psychol. (2005) 1:409–38. 10.1146/annurev.clinpsy.1.102803.144029 17716094

[7] Herringa RJ Birn RM Ruttle PL Burghy CA Stodola DE Davidson RJ. Childhood maltreatment is associated with altered fear circuitry and increased internalizing symptoms by late adolescence. Proc Natl Acad Sci U S A. (2013) 110:19119–24. 10.1073/pnas.1310766110 24191026 PMC3839755

[8] Vos T Lim SS Abbafati C Abbas KM Abbasi M Abbasifard M. Global burden of 369 diseases and injuries in 204 countries and territories, 1990–2019: a systematic analysis for the Global Burden of Disease Study 2019. Lancet. (2020) 396:1204–22. 10.1016/S0140-6736(20)30925-9 33069326 PMC7567026

[9] Klumparendt A Nelson J Barenbrügge J Ehring T. Associations between childhood maltreatment and adult depression: a mediation analysis. BMC Psychiatry. (2019) 19:36. 10.1186/s12888-019-2016-8 30669984 PMC6343339

[10] Nanni V Uher R Danese A. Childhood maltreatment predicts unfavorable course of illness and treatment outcome in depression: a meta-analysis. Am J Psychiatry. (2012) 169:141–51. 10.1176/appi.ajp.2011.11020335 22420036

[11] Li M D'Arcy C Meng X. Maltreatment in childhood substantially increases the risk of adult depression and anxiety in prospective cohort studies: systematic review, meta-analysis, and proportional attributable fractions. Psychol Med. (2016) 46:717–30. 10.1017/S0033291715002743 26708271

[12] Comijs HC Van Exel E Van Der Mast RC Paauw A Oude Voshaar R Stek ML. Childhood abuse in late-life depression. J Affect Disord. (2013) 147:241–6. 10.1016/j.jad.2012.11.010 23196199

[13] Bernet CZ Stein MB. Relationship of childhood maltreatment to the onset and course of major depression in adulthood. Depress Anxiety. (1999) 9:169–74. 10431682

[14] Norman RE Byambaa M De R Butchart A Scott J Vos T. The long-term health consequences of child physical abuse, emotional abuse, and neglect: a systematic review and meta-analysis. PLoS Med. (2012) 9:e1001349. 10.1371/journal.pmed.1001349 23209385 PMC3507962

[15] Nelson J Klumparendt A Doebler P Ehring T. Childhood maltreatment and characteristics of adult depression: meta-analysis. Br J Psychiatry. (2017) 210:96–104. 10.1192/bjp.bp.115.180752 27908895

[16] Beck AT Rush AJ Shaw BF EG. Cogn Ther Depress. New York, NY: Guildf Press (1979).

[17] Ezawa ID Forand NR Strunk DR. An examination of dysfunctional attitudes and extreme response styles as predictors of relapse in guided internet-based cognitive behavioral therapy for depression. J Clin Psychol. (2020) 76:1047–59. 10.1002/jclp.22955 32319092 PMC7319255

[18] Pedrelli P Feldman GC Vorono S Fava M Petersen T. Dysfunctional attitudes and perceived stress predict depressive symptoms severity following antidepressant treatment in patients with chronic depression. Psychiatry Res. (2008) 161:302–8. 10.1016/j.psychres.2007.08.004 18976817

[19] Akbaba Turkoglu S Essizoglu A Kosger F Aksaray G. Relationship between dysfunctional attitudes and childhood traumas in women with depression. Int J Soc Psychiatry. (2015) 61:796–801. 10.1177/0020764015585328 25977359

[20] Peng H Long Y Li J Guo Y Wu H Yang YL. Hypothalamic-pituitary-adrenal axis functioning and dysfunctional attitude in depressed patients with and without childhood neglect. BMC Psychiatry. (2014) 14:1–7. 10.1186/1471-244X-14-45 24548345 PMC3937002

[21] Abramson LY Metalsky GI Alloy LB. Hopelessness depression: a theory-based subtype of depression. Psychol Rev. (1989) 96:358–72. 10.1037/0033-295X.96.2.358

[22] Abramson LY. Cognitive/personality subtypes of depression: theories in search of disorders. Cognit Ther Res. (1997) 21:247–65. 10.1023/A:1021870315058

[23] Hamilton M. A rating scale for depression. J Neurol Neurosurg Psychiatry. (1960) 23:56–62. 10.1136/jnnp.23.1.56 14399272 PMC495331

[24] Tang Y Zhang M. HAMD. Shanghai Ment Heal Cent. (1984) 4:9–12.

[25] Hamilton M. The assessment of anxiety states by rating. Br J Med Psychol. (1959) 32:50–5. 10.1111/j.2044-8341.1959.tb00467.x 13638508

[26] Wang C Chu Y Zhang Y Zhang N Zhang J Yang H. Study on factor structure of hamilton rating scale for anxiety. J Clin Psychiatry. (2011) 21:299–301.

[27] Weissman AN Beck AT. Development and validation of the dysfunctional attitude scale a preliminary investigation. In: The 62nd Annual Meeting of the American Educational Research Association; 1978 March 27–31 (Toronto, ON) (1978).

[28] Chen Y Xu J Yan S Xian Y Li Y Chang X. A study of the dysfunction attitude scale. China Chinese Ment Heal. (1998) 12:265–8.

[29] Wong DFK Kin SC Lau Y. The reliability and validity of the Chinese version of the dysfunctional attitudes scale form a (DAS-A) in a community sample. Int J Psychiatry Med. (2008) 38:141–52. 10.2190/PM.38.2.b 18724566

[30] Liu B Sun J Qin X Wang M Lu X Dong Q. State-dependent and trait-like characteristics of dysfunctional attitudes in patients with major depressive disorder. Front Psychiatry. (2020) 11:1. 10.3389/fpsyt.2020.00645 32754060 PMC7381299

[31] Qin X Sun J Wang M Lu X Dong Q Zhang L. Gender differences in dysfunctional attitudes in major depressive disorder. Front Psychiatry. (2020) 11:86. 10.3389/fpsyt.2020.00086 32180737 PMC7057763

[32] Bernstein DP Fink L Handelsman L Foote J Lovejoy M Wenzel K. Initial reliability and validity of a new retrospective measure of child abuse and neglect. Am J Psychiatry. (1994) 151:1132–6. 10.1176/ajp.151.8.1132 8037246

[33] Bernstein DP Stein JA Newcomb MD Walker E Pogge D Ahluvalia T. Development and validation of a brief screening version of the Childhood Trauma Questionnaire. Child Abus Negl. (2003) 27:169–90. 10.1016/S0145-2134(02)00541-0 12615092

[34] Fu WQ Yao S-Q. Initial reliability and validity of Childhood Trauma Questionnaire (CTQ-SF) applied in Chinese college students. Chin J Clin Psychol. (2005) 13:40–2. 10.16128/j.cnki.1005-3611.2005.01.012

[35] Howell DC. Statistical Methods for Psychology. 7th ed. Belmont, CA: Wadsworth, Cengage Learning (2010).

[36] Fox J. Applied regression analysis and generalized linear models. 2nd ed. Thousand Oaks, CA: SAGE Publications (2008).

[37] Maxwell SE Delaney HD. Designing Experiments and Analyzing Data: A Model Comparison Perspective. 2nd ed. New York, NY: Psychology Press (2004).

[38] Kinnear PR Gray CD. PASW 17 Statistics Made Simple. New York, NY: Psychology Press (2010).

[39] Keppel G Wickens TD. Design and Analysis: A Researcher's Handjournal. 4th ed. Upper Saddle River, NJ: Prentice Hall (2004).

[40] Jaccard J. Interaction Effects in Factorial Analysis of Variance. Thousand Oaks, CA: SAGE Publications, Inc. (1998).

[41] Beck AT. The evolution of the cognitive model of depression and its neurobiological correlates. Am J Psychiatry. (2008) 165:969–77. 10.1176/appi.ajp.2008.08050721 18628348

[42] Miranda J Gross JJ Persons JB Hahn J. Mood matters: negative mood induction activates dysfunctional attitudes in women vulnerable to depression. Cognit Ther Res. (1998) 22:363–76. 10.1023/A:1018709212986

[43] Wells TT Vanderlind WM Selby EA Beevers CG. Childhood abuse and vulnerability to depression: cognitive scars in otherwise healthy young adults. Cogn Emot. (2014) 28:821–33. 10.1080/02699931.2013.864258 24313549 PMC4214063

[44] Rose DT Abramson LY. Developmental predictors of depressive cognitive style: research and theory. Rochester Symp Dev Psychopathol. (1992) 4:323–49.29368955

[45] Alloy LB Abramson LY Smith JM Gibb BE Neeren AM. Role of parenting and maltreatment histories in unipolar and bipolar mood disorders: mediation by cognitive vulnerability to depression. Clin Child Fam Psychol Rev. (2006) 9:23–64. 10.1007/s10567-006-0002-4 16718583

[46] Gibb BE Abramson LY Alloy LB. Emotional maltreatment from parents, verbal peer victimization, and cognitive vulnerability to depression. Cognit Ther Res. (2004) 28:1–21. 10.1023/B:COTR.0000016927.18027.c2

[47] Gibb BE. Childhood maltreatment and negative cognitive styles. Clin Psychol Rev. (2002) 22:223–46. 10.1016/S0272-7358(01)00088-5 11806020

[48] Maric N Andric S Mihaljevic M Mirjanic T Pavlovic Z. Sub-types of childhood trauma predicts depressive and anxiety symptoms in the general population. Eur Psychiatry. (2016) 33:S516. 10.1016/j.eurpsy.2016.01.1908

[49] Gibb BE Alloy LB Abramson LY Rose DT Whitehouse WG Donovan P. History of childhood maltreatment, negative cognitive styles, and episodes of depression in adulthood. Cognit Ther Res. (2001) 25:425–46. 10.1023/A:1005586519986

[50] Martins CMS Von Werne Baes C De Carvalho Tofoli SM Juruena MF. Emotional abuse in childhood is a differential factor for the development of depression in adults. J Nerv Ment Dis. (2014) 202:774–82. 10.1097/NMD.0000000000000202 25268154

[51] Stoltenborgh M Bakermans-Kranenburg MJ Van Ijzendoorn MH. The neglect of child neglect: a meta-analytic review of the prevalence of neglect. Soc Psychiatry Psychiatr Epidemiol. (2013) 48:345–55. 10.1007/s00127-012-0549-y 22797133 PMC3568479

[52] Teicher MH Dumont NL Ito Y Vaituzis C Giedd JN Andersen SL. Childhood neglect is associated with reduced corpus callosum area. Biol Psychiatry. (2004) 56:80–5. 10.1016/j.biopsych.2004.03.016 15231439

[53] Guo W Liu F Xue Z Gao K Wu R Ma C Liu Z. Altered white matter integrity in young adults with first-episode, treatment-naive, and treatment-responsive depression. Neurosci Lett. (2012) 522:139–44. 10.1016/j.neulet.2012.06.027 22721700

[54] Kemp A Macmaster FP Jaworska N Yang XR Pradhan S Mahnke D. Age of onset and corpus callosal morphology in major depression. J Affect Disord. (2013) 150:703–6. 10.1016/j.jad.2013.05.009 23769291

[55] Peng H Ning Y Zhang Y Yang H Zhang L He Z. White-matter density abnormalities in depressive patients with and without childhood neglect: a voxel-based morphometry (VBM) analysis. Neurosci Lett. (2013) 550:23–8. 10.1016/j.neulet.2013.06.048 23831354

[56] Finkelhor D Ormrod RK Turner HA. Poly-victimization: a neglected component in child victimization. Child Abus Negl. (2007) 31:7–26. 10.1016/j.chiabu.2006.06.008 17224181

[57] Finkelhor D Ormrod RK Turner HA Hamby SL. Measuring poly-victimization using the Juvenile Victimization Questionnaire. Child Abus Negl. (2005) 29:1297–312. 10.1016/j.chiabu.2005.06.005 16274741

[58] Negele A Kaufhold J Kallenbach L Leuzinger-Bohleber M. Childhood trauma and its relation to chronic depression in adulthood. Depress Res Treat. (2015) 2015:650804. 10.1155/2015/650804 26693349 PMC4677006

[59] Novelo M von Gunten A Gomes Jardim GB Spanemberg L Argimon II de L Nogueira EL. Effects of childhood multiple maltreatment experiences on depression of socioeconomic disadvantaged elderly in Brazil. Child Abuse Negl. (2018) 79:350–7. 10.1016/j.chiabu.2018.02.013 29522996

[60] Dovran A Winje D Arefjord K Tobiassen S Stokke K Skogen JC Øverland S. Associations between adverse childhood experiences and adversities later in life. Survey data from a high-risk Norwegian sample. Child Abuse Negl. (2019) 98:104234. 10.1016/j.chiabu.2019.104234 31689619

[61] Nemeroff CB Heim CM Thase ME Klein DN Rush AJ Schatzberg AF. Differential responses to psychotherapy versus pharmacotherapy in patients with chronic forms of major depression and childhood trauma. Proc Natl Acad Sci U S A. (2003) 100:14293–6. 10.1073/pnas.2336126100 14615578 PMC283585

